# Value of generic medicines: an health economics study

**DOI:** 10.1186/2052-3211-8-S1-P15

**Published:** 2015-10-05

**Authors:** Martin Albrecht, Ariane Höer, Anne Zimmermann

**Affiliations:** 1 IGES Institut GmbH, Berlin, 10117, Germany

## Background

Generic medicines provide an opportunity to obtain similar treatments at lower costs for patients and payers, while liberating budgets for financing new innovative medicines. The debate on generic medicines has been centered on affordability and cost-savings so far. Positive health impact of generic medicines has, however, been scarcely discussed. The aim of the study is to examine the value of generic medicines in a more comprehensive way, particularly including the patient-related value. This involves generic medicines' health impact in terms of not only medication adherence and compliance, but also in terms of health outcomes measured by primary endpoints or by more comprehensive health benefit measures as well as public health aspects.

## Methodology

The analysis is based primarily on a structured literature review in relevant literature databases with focus on medical and economic journals / references (e.g. Pubmed or Econlit). Additionally, an internet research is conducted to identify scientific reports and papers from influential stakeholders and institutions. For the research of literature regarding the clinical benefit, the focus is on relevant guidelines and recommendations which are usually based on an evidence-based literature review and thus reliably representing the current state of evidence. This structured review is conducted for three selected drug classes: antihypertensives, adjuvant endocrine therapy for breast cancer, and an- tidepressants.

## Results

The study is still ongoing and will be finished by the end of summer 2015. To this date, the following results can be shared: For the antihypertensives and adjuvant endocrine therapies to treat breast cancer, there is comprehensive evidence on the clinical benefit showing that patients benefit from drug treatment. Utilization of hypertension drugs and endocrine therapies in the last decades increased in the majority of the European countries. At the same time, hypertension-related mortality has decreased significantly in the EU countries (in Germany, for example, by about 50 % between 1998 and 2010). Breast cancer-related mortality decreased in European countries, in many of which by about a quarter up to a third. One of the designated causes for the observed mortality decline in both indications is better medicinal treatment options, though other factors such as guideline implementation also contributed. In a next step we will analyze the role of patient access to generic medicines as being the precondition for achieving both cost savings and health benefits.

## Conclusions

The conclusions are still pending. The study is ongoing but will be finished by end of summer 2015.

